# Core Items for a Standardized Resource Use Measure: Expert Delphi Consensus Survey

**DOI:** 10.1016/j.jval.2017.06.011

**Published:** 2018-06

**Authors:** Joanna C. Thorn, Sara T. Brookes, Colin Ridyard, Ruth Riley, Dyfrig A. Hughes, Sarah Wordsworth, Sian M. Noble, Gail Thornton, William Hollingworth

**Affiliations:** 1 School of Social and Community Medicine, University of Bristol, Bristol, UK; 2 Centre for Health Economics and Medicines Evaluation, Bangor Institute for Health and Medical Research, Bangor University, Bangor, UK; 3 Health Economics Research Centre, Nuffield Department of Population Health, University of Oxford, Oxford, UK

**Keywords:** cost measurement, patient-reported, randomized clinical trial, resource use

## Abstract

**Background:**

Resource use measurement by patient recall is characterized by inconsistent methods and a lack of validation. A validated standardized resource use measure could increase data quality, improve comparability between studies, and reduce research burden.

**Objectives:**

To identify a minimum set of core resource use items that should be included in a standardized adult instrument for UK health economic evaluation from a provider perspective.

**Methods:**

Health economists with experience of UK-based economic evaluations were recruited to participate in an electronic Delphi survey. Respondents were asked to rate 60 resource use items (e.g., medication names) on a scale of 1 to 9 according to the importance of the item in a generic context. Items considered less important according to predefined consensus criteria were dropped and a second survey was developed. In the second round, respondents received the median score and their own score from round 1 for each item alongside summarized comments and were asked to rerate items. A final project team meeting was held to determine the recommended core set.

**Results:**

Forty-five participants completed round 1. Twenty-six items were considered less important and were dropped, 34 items were retained for the second round, and no new items were added. Forty-two respondents (93.3%) completed round 2, and greater consensus was observed. After the final meeting, 10 core items were selected, with further items identified as suitable for “bolt-on” questionnaire modules.

**Conclusions:**

The consensus on 10 items considered important in a generic context suggests that a standardized instrument for core resource use items is feasible.

## Introduction

For cost-effectiveness analyses to be optimal, resource use measurement in randomized controlled trials (RCTs) must be accurate. Nevertheless, to date, considerably more research has been directed at improving outcome measurement methodologies (e.g., utilities) [1]. The methods used to measure costs are poorly reported [2], and instruments to collect data directly from patients are commonly not validated [3] (although there are studies in which the reliability/validity of self-report is considered [4]). When available, routine data sources (e.g., electronic hospital records) might reduce attrition bias, be more accurate, and minimize the burden on trial participants. Routine data may, however, not be readily available, consistent, or suitable for costing purposes [5]. Electronic systems may also be costly to access and may lack information on personal costs incurred by patients. It is therefore likely that researchers will continue to be reliant on instruments based on patient recall (e.g., diaries, logs, and questionnaires [6]) for some time, despite the fact that self-reported data on health care use are of variable accuracy [7].

A significant amount of work in recent years has focused on developing core outcome sets (COSs), which are agreed minimum sets of outcomes (often health-related) to be measured and reported in all trials for a specific condition/treatment [8]. Standardization counteracts problems with researchers selecting outcomes on the basis of their own expertise or the statistical significance of results. A standard set of outcomes also reduces heterogeneity and improves comparability across trials [9]. Although developing a core set of resource use items has much in common with COS development, there are also some important differences. A fundamental consideration of an economic analysis is the perspective, which leads to the inclusion of different types of resource use. Although COSs are specific to clinical conditions or treatments and are therefore different across trials, a core set of resource use is specific to the perspective, but could potentially be generalizable across trials. Separate measurement instruments may be required for outcomes identified in COSs (e.g., the EuroQol five-dimensional questionnaire for quality of life or the modified Health Assessment Questionnaire for patient satisfaction with activities of daily living [10]); in contrast, a core set of resource use items would generally form a single instrument.

Standardization of resource use measurement is potentially controversial among health economists. Legitimate concerns about the study perspective, nature of the intervention, and type of analysis planned may suggest that standardization is too limiting. There is a trade-off between gathering as much information as possible (with increased patient burden and possible poor response rates) and gathering less information (which may not allow an accurate analysis to be conducted). As Drummond et al. [11p253] point out, “The skill in costing is to match the level of precision (and effort) to the importance (in quantitative terms) of the cost item.” Nevertheless, standardizing outcomes using the EuroQol five-dimensional questionnaire is accepted in the United Kingdom (and indeed required by the National Institute for Health and Care Excellence [12]), despite the inevitable limitation on the flexibility of the instrument. In contrast, health economists typically generate new, or revise existing, resource use instruments for RCTs on a case-by-case basis; some standardization of cost measurement (albeit with “bolt-ons” to ensure more complete coverage of resources) would allow greater comparability between trials and would reduce the research effort required. The significant overlap between questions in instruments held in the Database of Instruments for Resource Use Measurement (www.dirum.org) [13] suggests that defining a core set may be feasible [14].

In our study (Items for a Standardised Resource-Use Measure, ISRUM), we aim to identify core items of resource use that should be included in any economic evaluation of a health care intervention conducted in the United Kingdom. We aim to identify a minimum set of items that should be measured, and not a complete set; we anticipate that health economists may measure additional items according to the particular nature of the RCT and the perspective of the analysis. We use a Delphi survey to seek consensus expert opinion.

## Methods

Approval for the study was granted by the Faculty of Health Sciences Research Ethics Committee of the University of Bristol. A patient and public involvement (PPI) representative was recruited to the study team via the People in Health West of England (http://www.phwe.org.uk/) mailing list.

### Phase 1: Identification of “Long List” and Development of Survey

The identification of a long list of resource use items is described in detail elsewhere [14]. In brief, a review of measurement instruments currently used in RCTs of health interventions was undertaken; individual items were extracted by two researchers and disagreements were resolved by discussion. Items were scrutinized by a single researcher and overlapping items merged. Similar types of items were combined; for example, doctor, nurse, and allied health professional were collapsed into “professional seen.” Items not relevant to a National Health Service (NHS) and personal social services (PSS) perspective (commonly taken in UK studies) were dropped. Remaining items were formulated as individual questions for a Delphi survey. The Delphi method is used increasingly for consensus in COSs [15]. It requires expert participants to provide their opinions in sequential questionnaires (rounds), with each round presenting group feedback from the previous round. Anonymity of the responses is maintained to ensure that no individual dominates the process [16]. A Web-administered “eDelphi” survey was developed using REDCap electronic data capture tools hosted at the University of Bristol [17]; items were grouped according to the location in which the care took place (e.g., hospital). The survey was piloted in the study team, and a think-aloud Web usability study (in which participants were asked to talk through their responses) was conducted with a convenience sample to ensure it was comprehensible and manageable [18].

### Phase 2: Prioritization of Resource Use Items

#### Stakeholders

Practicing health economists with experience of RCTs in the United Kingdom were recruited to the Delphi panel. A generic email was sent to the Health Economists’ Study Group mailing list describing the preparatory work and purpose of the study and inviting participation by following a Web link. Health economists who had recently contributed to National Institute for Health Research Health Technology Assessment reports (http://www.journalslibrary.nihr.ac.uk/hta) or attended relevant workshops were approached directly. One reminder email was sent. Completion of the first questionnaire was deemed to represent informed consent to participate. Demographic details were requested in the survey including subgroups describing experience with different types of patient care (physical, mental, and public health; older adults; primary and secondary care), length of experience, and professional background.

#### Survey round 1

In round 1 of the survey, participants were asked to rate the importance of retaining each item in the core standardized resource use set on a scale of 1 (not important) to 9 (very important). Participants were asked to think in terms of resource use relevant to an NHS and PSS perspective for adult patients of any age, living with wide-ranging physical and/or mental health conditions of variable severity (see Appendix 1 in Supplemental Materials found at doi:10.1016/j.jval.2017.06.011). They were asked to assume that there may be differences between trial arms in any item and that they have no access to any other source of resource use data (such as medical records). Participants were encouraged to comment on their ratings and suggest additional items. After completion of the questionnaire, items for which the participant had scored 7 to 9 were presented back to them, with a request to select their “top 10” items for the core set. Round 1 item scores were summarized across participants, and items to retain for round 2 were identified using prespecified criteria; items suggested by participants were added if they met prespecified criteria (see Analysis section).

#### Survey round 2

All participants who had completed round 1 of the survey were emailed a Web link to the round 2 questionnaire. Feedback from round 1 was presented for each round 2 item in the form of the median score along with a reminder of the individual’s own score. Comments in round 1 that were relevant to selection choice were also summarized and presented, and changes were made to the wording for a small number of items on the basis of some of the comments. Participants were asked to rerate each item (see Appendix 1 in Supplemental Materials) and were given further opportunity to comment on their choices. A reminder invitation was sent after 2 weeks, and a further reminder specifying a closing date was issued 1 week later. Shortly after the closing date, nonresponders were contacted by telephone to request reasons for noncompletion.

#### Analyses

Statistical analyses were carried out in Stata 14 (StataCorp LP, College Station, TX) [19] and were conducted according to a prespecified analysis plan.

#### Criteria for retaining items

At the end of round 1, the percentage of participants scoring 7 to 9 (high priority) and 1 to 3 (low priority) was calculated for each item, both for participants overall and for each of the “type of experience” subgroups separately. Items were retained if scored 7 to 9 by more than 50% and 1 to 3 by less than 15% by participants overall or within two or more subgroups of participants; these prespecified criteria were deliberately inclusive. Items were also retained if 15% or more of the participants prioritized the item in their top 10 list. Items not meeting any of these criteria were closely examined for overlap with retained items; if there was no overlap, the item was further considered for retention. New items were added to round 2 if suggested by more than 10% of participants.

After round 2, items were retained if scored 7 to 9 by more than 70% and 1 to 3 by less than 15% of all participants. Because further Delphi rounds were beyond the scope of this study, more stringent criteria were also set (>70% scoring an item 8 or 9 and <15% scoring 1–3) to aid discussions in a final item selection meeting so that a pragmatic core set could be identified.

#### Attrition

Nonresponders to round 2 were examined in terms of years of experience; mean scores were compared with those from round 2 responders.

#### Assessment of consensus

It is not a requirement of the Delphi process to achieve consensus for all items (e.g., when all participants agreed on the high/low priority grouping); it is, however, essential that participants agree on a reduced number of items to be most important. It is therefore informative to consider the level of agreement across participants in both rounds and the degree of stability in scores.

For each round, the percentage of participants scoring 7 to 9 and 1 to 3 was examined for evidence of bimodality (defined as >40% rating an item 7–9 and >40% rating it 1–3) for each item, because this could indicate an irreconcilable difference of opinion. The intraclass correlation coefficient (two-way random effects model) was calculated for both rounds, to give an indication of agreement within the survey [20].

For each item, the mean absolute change in score between rounds was also calculated; a large change (defined as ≥3 points) could indicate instability. The percentage of people changing their score by a small amount (1 or 2 points) and a large amount (≥3 points) was calculated for each item to give an indication of the stability of the results. Variation in changes to scores with length of experience (categorized as <5 years, 5–10 years, 10–20 years, and >20 years) was explored through linear regression. Finally, the SD of scores was calculated for each item (separately for each round) as a measure of the spread in responses across participants (and degree of agreement) and was used to calculate the change in each item’s variability between rounds [21].

#### Analysis of comments

Content analysis (a systematic approach to studying text that aims to categorize and quantify content) was conducted for comments by using nVivo software (QSR International Ltd. London) [22], [23]. Suggestions in round 1 for new items were extracted, and broad themes were identified for both rounds.

### Phase 3: Final Item Selection Meeting

The project team met to determine the final core items to include in a standardized “short form” resource use measure. Participants who had commented extensively during the Delphi process or were associated with the Medical Research Council (MRC) Network of Hubs for Trials Methodology Research were invited to attend the meeting. Each item included in round 2 was discussed in detail. The two prespecified criteria were applied to the round 2 data to identify the items considered most crucial (more stringent criteria) and very important (less stringent criteria) for inclusion in the final core set. Items reaching the more stringent criteria were included in the final set if considered relevant, by the team, to all trials and patient populations. If relevant only to specific settings, items were included in suggested bolt-on modules. Items reaching the less stringent criteria were then discussed and merged with those already in the final set when appropriate or were considered as separate items for the core set or as items in bolt-on modules. Remaining items were examined to ensure that nothing vital was overlooked.

## Results

### Phase 1

Items were extracted from 59 resource use instruments. After the deduplication and merging processes, the long list contained 60 items, categorized as hospital care (n = 15), emergency care (n = 5), care at a general practitioner (GP) surgery or health clinic (n = 7), care at home (n = 7), remote access care (n = 4), other community care (n = 6), residential care (n = 10), and medication (n = 6). Usability studies with both a native and a non-native English speaker indicated that the Delphi survey was comprehensible, and completion was manageable.

### Phase 2

**Fig. 1:**
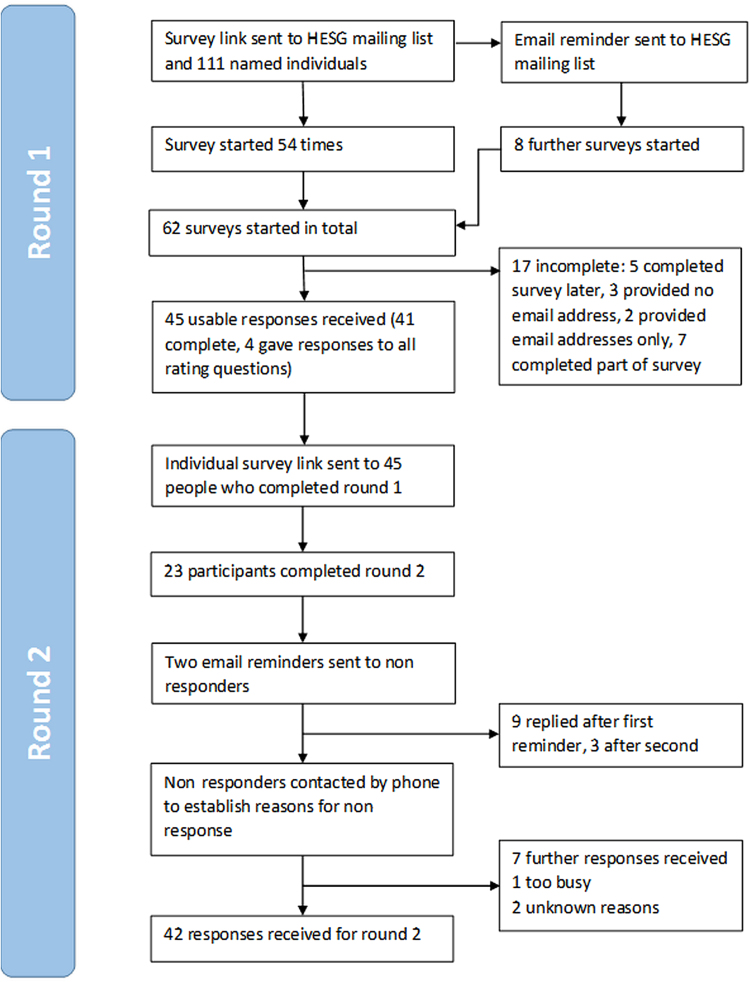
Flowchart of Delphi study participants through the study. HESG, Health Economists’ Study Group.

Forty-five participants provided usable responses to round 1; 41 completed the whole survey, whereas 4 supplied ratings for all items, but did not select their top 10 (Fig. 1). Participants with a range of experience were represented (Table 1), although almost all (42 of 45) were working in academia. Application of the predefined consensus criteria identified 27 items to be retained for round 2, considered to be of high priority by participants overall. Four additional items were considered important by two or more subgroups: minor surgery (important to participants with experience of primary care, physical health, public health, or older adults), living in either a residential home or a supported accommodation (rated highly by participants with experience of primary care, mental health, or older adults), and the period over which medication is taken (important to respondents with experience in primary care and public health). Type of ward and scans were added because more than 15% of respondents cited them in their top 10. Finally, equipment was identified as a suitable addition because it came close to meeting several of the aforementioned criteria and no other similar items were included. No new items met the inclusion criteria. Thirty-four items were therefore included for round 2 (Table 2) and 26 items were dropped (Table 3). Engagement with the project in round 1 was good, with broadly positive comments indicating that achieving consensus was feasible.

**Table 1:** Characteristics of 45 Delphi participants from round 1

**Characteristic**	**n (%)**
Length of experience	
<5 y	11 (24.4)
5–10 y	12 (26.7)
10–20 y	11 (24.4)
>20 y	11 (24.4)
Trial experience	
Adults	44 (97.8)
Children	21 (46.7)
Older adults	26 (57.8)
Physical health conditions	38 (84.4)
Mental health conditions	28 (62.2)
Public health interventions	20 (44.4)
Primary care	33 (73.3)
Secondary care	39 (86.7)
Background	
Academia	42 (93.3)
Other	3 (6.7)

**Table 2:** Items retained at the end of round 1

**Item description**	**% rating 7–9**	**% rating 1–3**	**Median (IQR)**	**Inclusion reason**
*Hospital care*				
(1) Number of hospital admissions (inpatient stay or day case)	95.56	0.00	9 (9–9)	Consensus
(2) Number of hospital outpatient appointments	91.11	2.22	9 (8–9)	Consensus
(3) Length of stay (e.g., dates or number of nights)	84.44	0.00	9 (8–9)	Consensus
(4) Number of operations/procedures undergone	64.44	4.44	8 (6–9)	Consensus
(5) Type of operation/procedure undergone	64.44	4.44	8 (6–9)	Consensus
(6) Type of professional seen (e.g., consultant/nurse)	53.33	13.33	7 (5–8)	Consensus
(7) Number of imaging scans undergone (e.g., x-ray/MRI)	42.22	13.33	6 (5–8)	Top 10*
(8) Type of ward stayed in	37.78	11.11	6 (5–8)	Top 10*
*Emergency care*				
(9) Number of visits to A&E	91.11	0.00	9 (7–9)	Consensus
(10) Number of admissions to hospital, after A&E	80.00	2.22	9 (7–9)	Consensus
(11) Number of times paramedic care received	53.33	4.44	7 (5–9)	Consensus
*Care at a GP surgery or health clinic*				
(12) Number of appointments at a GP surgery or health clinic	95.56	2.22	9 (9–9)	Consensus
(13) Type of professional seen (e.g., GP/nurse/counselor)	80.00	0.00	9 (7–9)	Consensus
(14) Number of minor surgery/procedures/treatments undergone	46.67	6.67	6 (5–9)	Subgroup†
*Care at home*				
(15) Number of health care or social care professional visits at home (e.g., health visitor/GP)	86.67	2.22	9 (8–9)	Consensus
(16) Type of professional seen at home	80.00	0.00	9 (7–9)	Consensus
(17) Number of professional visits for help with daily activities (e.g., washing/dressing)	55.56	6.67	7 (5–9)	Consensus
(18) Equipment (e.g., wheelchairs/portable oxygen/specialist clothing) or home adaptation (e.g., grab rails/ramp) supplied	44.44	13.33	6 (5–8)	Close‡
*Remote access care*				
(19) Number of real-time telephone/computer contacts with health or social care professional (e.g., with GP or telephone helpline)	68.89	4.44	7 (6–9)	Consensus
(20) Type of professional contacted (e.g., doctor/nurse/social worker)	53.33	6.67	7 (5–9)	Consensus
*Other community care*				
(21) Number of visits to health care professional in the community (e.g., dentist, pharmacist, nurse, counselor, and therapist)	80.00	2.22	9 (7–9)	Consensus
(22) Number of visits to social care professional in the community (e.g., social worker/housing worker/drug and alcohol worker)	75.56	2.22	9 (7–9)	Consensus
(23) Type of health care professional seen	71.11	4.44	8 (5–9)	Consensus
(24) Type of social care professional seen in the community	62.22	4.44	8 (5–9)	Consensus
*Residential care*				
(25) Stay in hospice	77.78	6.67	9 (7–9)	Consensus
(26) Length of time spent in the hospice	75.56	8.89	8 (7–9)	Consensus
(27) Use of short-term respite or rehabilitation care	66.67	6.67	7 (5–9)	Consensus
(28) Length of stay in short-term respite or rehabilitation care	62.22	8.89	7 (5–9)	Consensus
(29) Living in a nursing home	53.33	11.11	7 (5–9)	Consensus
(30) Living in a residential home	48.89	11.11	6 (5–9)	Subgroup†
(31) Living in supported accommodation/sheltered housing	46.67	11.11	6 (5–9)	Subgroup†
*Medication*				
(32) Number of prescribed medications	68.89	6.67	8 (5–9)	Consensus
(33) Name of medication	64.44	4.44	8 (5–9)	Consensus
(34) Period taken for (e.g., dates or number of days)	46.67	11.11	6 (5–9)	Subgroup†

A&E, accident and emergency; GP, general practitioner; IQR, interquartile range; MRI, magnetic resonance imaging.

⁎ Item included because >15% of participants listed it in their “top 10” choices.

† Item included because >1 subgroup rated it highly.

‡ Item included because it came close to meeting several criteria.

**Table 3:** Items dropped at the end of round 1

**Item description**	**% rating 7**–**9**	**% rating 1**–**3**	**Median (IQR)**
*Hospital care*			
Number of other procedures undergone	35.56	20.00	6 (4–7)
Number of laboratory tests undergone	35.56	24.44	5 (4–7)
Type of imaging scans undergone	31.11	15.56	6 (5–8)
Type of other procedures undergone	26.67	17.78	5 (4–7)
Type of laboratory tests undergone	22.22	26.67	5 (3–6)
Length of outpatient appointment	15.56	55.56	3 (2–5)
Number of hospital transport journeys (nonemergency)	13.33	42.22	5 (2–5)
*Emergency care*			
Number of ambulance journeys	28.89	17.78	5 (4–7)
Time spent in A&E	15.56	46.67	4 (3–5)
*Care at a GP surgery or health clinic*			
Number of laboratory tests undergone	40.00	22.22	5 (4–7)
Type of minor surgery/procedures/treatments undergone	33.33	11.11	5 (5–7)
Timing of appointments (office hours or out of hours)	33.33	31.11	5 (3–9)
Type of laboratory tests undergone	26.67	28.89	5 (3–7)
*Care at home*			
Type of equipment or adaptation supplied	35.56	22.22	5 (4–7)
Time spent with professional at home	33.33	22.22	5 (4–7)
Time spent by professional for help with daily activities	31.11	15.56	5 (5–7)
*Remote access care*			
Duration of contact with professional	24.44	33.33	5 (3–6)
Number of email or SMS (text) communications with health care professional	13.33	42.22	4 (3–5)
*Other community care*			
Use of patient support services in the community (e.g., self-help groups/lunch clubs/day center)	28.89	15.56	5 (4–7)
Type of support service used	24.44	22.22	5 (4–6)
*Residential care*			
Date moved to nursing home	46.67	13.33	6 (5–9)
Date moved to residential home	42.22	13.33	6 (5–9)
Date moved to supported accommodation/sheltered housing	40.00	13.33	5 (5–9)
*Medication*			
Frequency taken	42.22	15.56	6 (5–9)
Dose taken	42.22	15.56	5 (5–9)
Route taken (e.g., oral/suppository/intravenous)	24.44	35.56	5 (3–6)

A&E, accident and emergency; GP, general practitioner; IQR, interquartile range.

Out of 45 participants, 42 (93.3%) responded to round 2 (Fig. 1). The three nonresponders each came from a different level of experience. Nonresponders had a mean score of 8.53 ± 0.33 in round 1 compared with 7.13 ± 1.09 for responders (*P* = 0.03). There was no evidence of bimodality for any item in either round. All responding participants changed at least one rating between rounds, and all items were changed by at least one participant. Participants changed their scores by a mean of 0.70 ± 0.36 points between rounds.

The intraclass correlation coefficient (95% confidence interval) increased from 0.85 (0.77–0.91) in round 1 to 0.93 (0.89–0.96) in round 2, suggesting increased consensus in round 2. Between rounds, SDs reduced for all individual items except for hospital admission items and prescribed medication (Table 4), again suggesting movement toward increased consensus in round 2. As anticipated, 100% concordance on the priority group (high/low) was not achieved for any item in either round. No relationship was observed between changes to mean scores and length of experience.

**Table 4:** Indicators of response to feedback from round 1

**Item description**	**Mean ± SD**		**Round 2 − Round 1**		**% rating an item 7**–**9**		**% changing score by**	
	**Round 1**	**Round 2**	**Change in mean**	**Change in SD**	**Round 1**	**Round 2**	**1 or 2**	**≥3**
GP appointments	8.5 ± 1.1	8.7 ± 0.9	0.13	−0.23	95.56	97.62	21.43	0.00
GP surgery/procedure	6.3 ± 2.1	5.8 ± 1.9	−0.52	−0.25	46.67	35.71	52.38	2.38
GP professional seen	7.9 ± 1.5	8.2 ± 1.2	0.30	−0.35	80.00	92.86	23.81	2.38
Equipment	6.1 ± 2.2	6.0 ± 1.7	−0.13	−0.48	44.44	33.33	45.24	4.76
Home visits	8.3 ± 1.3	8.5 ± 0.8	0.19	−0.49	86.67	97.62	11.90	2.38
Help with activities	6.9 ± 2.1	6.8 ± 1.5	−0.13	−0.58	55.56	59.52	59.52	4.76
Professional seen at home	7.8 ± 1.6	8.0 ± 1.4	0.20	−0.23	80.00	88.10	21.43	7.14
Admissions after A&E	7.8 ± 1.7	7.9 ± 1.8	0.06	0.06	80.00	88.10	21.43	16.67
Paramedic care	6.8 ± 2.0	6.7 ± 1.3	−0.07	−0.68	53.33	50.00	52.38	7.14
A&E	8.2 ± 1.2	8.5 ± 0.8	0.25	−0.40	91.11	95.24	26.19	2.38
Length of stay	8.2 ± 1.5	8.4 ± 1.4	0.20	−0.02	84.44	92.86	23.81	2.38
Hospital admissions	8.7 ± 0.8	8.6 ± 1.2	−0.14	0.38	95.56	97.62	9.52	2.38
Hospital outpatients	8.2 ± 1.5	8.5 ± 0.9	0.21	−0.52	91.11	97.62	26.19	2.38
Imaging scans	6.2 ± 2.3	5.6 ± 1.8	−0.58	−0.51	42.22	23.81	33.33	9.52
Operation/procedure	7.2 ± 2.1	7.5 ± 1.4	0.28	−0.75	64.44	78.57	45.24	7.14
Specialty/ward	6.2 ± 2.0	6.1 ± 1.7	−0.08	−0.26	37.78	38.10	45.24	7.14
Operation type	7.1 ± 2.0	7.3 ± 1.4	0.17	−0.59	64.44	71.43	35.71	7.14
Professional seen outpatient	6.2 ± 2.2	6.4 ± 1.7	0.16	−0.49	53.33	57.14	47.62	7.14
Name of medication	7.1 ± 2.1	7.3 ± 1.9	0.17	−0.20	64.44	73.81	42.86	14.29
Prescribed medication	7.2 ± 2.3	6.7 ± 2.3	−0.51	0.03	68.89	64.29	35.71	9.52
Period taken for	6.4 ± 2.3	6.3 ± 1.8	−0.09	−0.41	46.67	40.48	42.86	9.52
Community health care	7.8 ± 1.7	8.1 ± 1.3	0.29	−0.41	80.00	88.10	33.33	4.76
Community social care	7.7 ± 1.8	8.0 ± 1.2	0.22	−0.55	75.56	85.71	35.71	4.76
Health professional seen	7.2 ± 2.0	7.5 ± 1.5	0.30	−0.56	71.11	78.57	33.33	7.14
Social care professional seen	7.0 ± 2.2	7.4 ± 1.6	0.38	−0.56	62.22	73.81	35.71	9.52
Telephone/computer contacts	7.2 ± 1.9	7.0 ± 1.5	−0.27	−0.43	68.89	64.29	50.00	2.38
Professional contacted	6.6 ± 2.0	6.8 ± 1.5	0.16	−0.51	53.33	54.76	52.38	2.38
Respite length of stay	6.9 ± 2.3	6.7 ± 1.9	−0.24	−0.36	62.22	64.29	57.14	2.38
Hospice length of stay	7.4 ± 2.1	7.4 ± 2.1	−0.02	−0.02	75.56	78.57	33.33	7.14
Nursing home	6.8 ± 2.4	6.6 ± 2.0	−0.14	−0.42	53.33	57.14	57.14	4.76
Residential home	6.7 ± 2.4	6.2 ± 2.1	−0.50	−0.28	48.89	40.48	42.86	11.90
Supported accommodation	6.4 ± 2.5	5.9 ± 2.0	−0.52	−0.52	46.67	30.95	40.48	9.52
Stay in hospice	7.5 ± 2.1	7.6 ± 2.1	0.13	−0.05	77.78	80.95	19.05	11.90
Respite care	7.0 ± 2.2	6.9 ± 1.9	−0.16	−0.32	66.67	73.81	47.62	7.14

A&E, accident and emergency; GP, general practitioner.

Twenty-eight respondents commented in round 1, with two not completing the survey. The content analysis showed that the hospital and home care categories attracted the highest number of comments (15 and 11, respectively). Some comments indicated that the task was cognitively challenging. The most common theme was that the inclusion of a particular item depended on another factor including perspective, intervention, setting, condition, patient group, level of detail, recall period, time horizon, and comparator. Potential issues with patient recall and practical aspects of administering a resource use questionnaire were also raised. Seventeen respondents commented in round 2; comments largely focused on useful suggestions for developing an instrument, with seven individuals suggesting a modular approach.

### Phase 3

In addition to the project team, three Delphi participants were invited to attend the final item selection meeting; because of other commitments, only one was available. The selection group identified community health care questions that could be combined with GP questions for consistency. Items asking about details of hospital operations or procedures were considered less important by the more stringent set of consensus rules and were rejected for the core set of items for the short form (Table 5). These items could be included in an extended hospital care module for trials in which admissions (or re-admissions) for procedures are prevalent. Similarly, most residential care items (with the exception of hospice stays) did not meet the stringent consensus rules. Although residential care was thought to be extremely important in some trials, it was judged by the selection meeting group to be not relevant in most trials and was therefore identified as a suitable candidate for a bolt-on module. Items on social care did not meet the more stringent consensus rules, potentially because they were considered to be more relevant to particular groups, such as older adults; these items could therefore be included in a bolt-on social care module. Perhaps surprisingly, items on medication use were not identified as important by the more stringent criterion rules. The selection committee group felt that medication use was relevant to participants in most of the trials and should therefore remain on the included list; nevertheless, future work will look at the practical aspects of collecting medication data, and medication may form a separate module in the future.

**Table 5:** Final outcomes for items after round 2

**Item description**	**% rating 7–9**	**% rating 8 or 9**	**% rating 1–3**	**Outcome: pre-agreed rules**	**Outcome: more stringent rules**	**Final outcome after item selection meeting**
Hospital outpatients	97.62	80.95	0.00	Include	Include	Short form
Hospital admissions	97.62	90.48	2.38	Include	Include	Short form
Length of stay	92.86	85.71	2.38	Include	Include	Short form
Operation/procedure	78.57	61.90	0.00	Include	Exclude	Extended hospital care module
Operation type	71.43	54.76	0.00	Include	Exclude	Extended hospital care module
A&E	95.24	88.10	0.00	Include	Include	Short form
Admissions after A&E	88.10	76.19	7.14	Include	Include	Short form
GP appointments	97.62	95.24	0.00	Include	Include	Short form
GP professional seen	92.86	78.57	0.00	Include	Include	Short form
Home visits	97.62	85.71	0.00	Include	Include	Short form
Professional seen at home	88.10	69.05	2.38	Include	Exclude	Short form
Community health care	88.10	78.57	2.38	Include	Include	Combined with GP appointments in short form
Community social care	85.71	69.05	0.00	Include	Exclude	Social care module
Health professional seen	78.57	57.14	2.38	Include	Exclude	Combined with GP appointments in short form
Social care professional seen	73.81	52.38	2.38	Include	Exclude	Social care module
Stay in hospice	80.95	73.81	9.52	Include	Include	Residential care module
Hospice length of stay	78.57	66.67	11.90	Include	Exclude	Residential care module
Respite care	73.81	40.48	7.14	Include	Exclude	Residential care module
Name of medication	73.81	59.52	4.76	Include	Exclude	Short form
Professional seen outpatient	57.14	26.19	4.76	Exclude	Exclude	
Specialty/ward	38.10	16.67	9.52	Exclude	Exclude	
Imaging scans	23.81	11.90	9.52	Exclude	Exclude	
Paramedic care	50.00	23.81	0.00	Exclude	Exclude	
GP surgery/procedure	35.71	16.67	9.52	Exclude	Exclude	
Help with activities	59.52	30.95	2.38	Exclude	Exclude	
Equipment	33.33	21.43	2.38	Exclude	Exclude	
Telephone/computer contacts	64.29	38.10	2.38	Exclude	Exclude	
Professional contacted	54.76	35.71	2.38	Exclude	Exclude	
Respite length of stay	64.29	40.48	7.14	Exclude	Exclude	
Nursing home	57.14	35.71	9.52	Exclude	Exclude	
Residential home	40.48	28.57	11.90	Exclude	Exclude	
Supported accommodation	30.95	19.05	11.90	Exclude	Exclude	
Prescribed medication	64.29	47.62	14.29	Exclude	Exclude	
Period taken for	40.48	28.57	4.76	Exclude	Exclude	

A&E, accident and emergency; GP, general practitioner.

## Discussion

On the basis of consensus among health economists, we have identified a minimum core set of 10 resource use items that should be considered for inclusion in a standardized questionnaire for patients (Table 6). We have identified additional items that are suitable for inclusion as bolt-on or extended modules covering further details about hospital procedures, residential care, and social care. Agreement among participants was excellent [24] and moved toward consensus in the second round. Results were reasonably stable, suggesting that a third round would not have significantly altered the outcome. Although the survey was conducted from the viewpoint of the NHS and PSS, the key inclusions are all items commonly provided by the NHS. Social services care could therefore form a separate bolt-on module for trial populations in which it is thought to be prevalent.

**Table 6:** Items included in the final core set

**Type of care**	**Item**
1. Hospital care	Number of hospital admissions (inpatient stay or day case)
2. Hospital care	Length of stay (e.g., dates or number of nights)
3. Hospital care	Number of hospital outpatient appointments
4. Emergency care	Number of visits to A&E
5. Emergency care	Number of admissions to hospital, after A&E
6. Care at a GP surgery or health clinic or other community setting	Number of appointments
7. Care at a GP surgery or health clinic or other community setting	Type of professional seen
8. Health care at home	Number of health care professional visits at home
9. Health care at home	Type of health care professional seen at home
10. Medication	Name/class of medication

A&E, accident and emergency; GP, general practitioner.

Knapp and Beecham [25] identified “reduced lists” of key services that could be measured to capture over 90% of the total costs of health and social care in patient groups with mental health conditions. The study indicated that, in principle, capturing a fairly small number of key items of resource use can lead to adequate cost information, with diminishing returns gained by further data collection. Nevertheless, although there was some overlap with the items we identified in this study (hospital inpatient and outpatient, residential care, and GP care), the nature of the patient group meant that social services played a considerably more prominent role.

Generic resource use measures developed to date include the Annotated Patient Cost Questionnaire [26] and the Client Service Receipt Inventory [27]. The former was designed as a generic patient-reported instrument. Although empirical evidence suggests that the questionnaire performs well [28], it has not been widely adopted (possibly because of the length of the questionnaire necessitating substantial work to generate an instrument for a trial). The latter has been tested extensively, demonstrating good consistency, reliability, and validity [29], [30], [31], [32] and is well used. Nevertheless, it was developed in the context of psychiatric care, was designed for interview administration rather than patient self-completion, and has been subject to uncontrolled modification over the years. Standardization of data collection has also been attempted in the context of cancer care [33], and a generic Dutch language instrument has been developed [34]. Nevertheless, neither implementation combines full standardization across all disease areas with a concise instrument and neither attempts to determine relevant content through a documented consensus process involving health economists.

Strengths of the study include the recruitment of the panel of expert participants, who were representative of a wide range of experience and had extensive NHS research experience. The stability of the panel was good with less than 10% attrition, and the study benefited from patient involvement in the study team. Established methods for conducting Delphi surveys were followed, with consensus criteria defined in advance of conducting each round. There was clear consensus for items ultimately included in the core set. Nevertheless, there may also be some limitations. Almost all the respondents came from an academic background; wider participation from industry representatives may have been beneficial in terms of generalizability, although their experience of NHS research would have been more limited. A larger sample participating in the Delphi survey would have been preferable; there is, however, no statistical basis on which to determine necessary sample size for a Delphi survey, and previous studies including fewer participants have been shown to produce reliable results [35]. Respondents were asked to rate the type of resource use (e.g., hospital or GP care) as well as the measurement information (such as the number of nights or appointments) simultaneously. The task was therefore cognitively challenging, with a large set of factors to bear in mind while responding; it is possible that participants may not have taken everything relevant into account.

The items identified are those considered most important by professional health economists for inclusion in a core set of resource use items. Work is now needed to identify the most appropriate way to measure these items to ensure patient acceptability and comprehensibility. There was evidence from the comments that some participants were considering patient ability to respond to questions. For example, one respondent commented that “… many patient groups are very confused about which services and professionals have visited them at home.” This requires further investigation with patient groups. Patients were not recruited to the Delphi panel, because the task was not meaningful in the context of the UK health care system in which patients do not pay for services at the point of use. The patient perspective was, however, represented during the study by the PPI member of the project team. Translation of the questionnaire to other languages (and other health care systems) also requires further investigation; given the common nature of the items included, it is possible that it will extend readily to other health care systems.

In this project, we have focused on an NHS and PSS perspective. There will commonly be requirements for additional data to be collected; any future instrument should take this into account through modularization, allowing modifications in a controlled fashion only, with alterations recorded. It is also likely that the resource use associated with the intervention itself will need to be collected separately. The developed instrument should be reviewed regularly to ensure that it remains current; for example, remote access care does not feature in our short form, but may become more pertinent in future if online consultations become common. We plan to develop a core module based on the 10 items identified in this study, working with PPI representatives to convert the items into questions that are meaningful and straightforward to answer.

## Conclusions

The consensus on which items are important to health economists working on clinical trials in a generic context suggests that a standardized instrument for core items is feasible. The list of items identified forms a coherent set that is potentially relevant to most trials, conditions, and patient groups; it is therefore suitable for further development into a flexible instrument with additional extended and bolt-on modules. Collecting cost data in a manner that is simultaneously concise, understandable for patients, valid, precise, consistent between trials, and generalizable is challenging. We have provided much needed evidence that it may be possible to develop a standardized instrument that goes some way to meeting those challenges, on the basis of the most important cost items.
