# Supplementary material for: Core Items for a Standardized Resource Use Measure: Expert Delphi Consensus Survey
Source: Value Health. 2018 Jun;21(6):640–9. doi: 10.1016/j.jval.2017.06.011 (PMC6021557; doi:10.1016/j.jval.2017.06.011)

Appendix 1. Delphi survey instructions

**Figure A1**. Instructions for round 1.


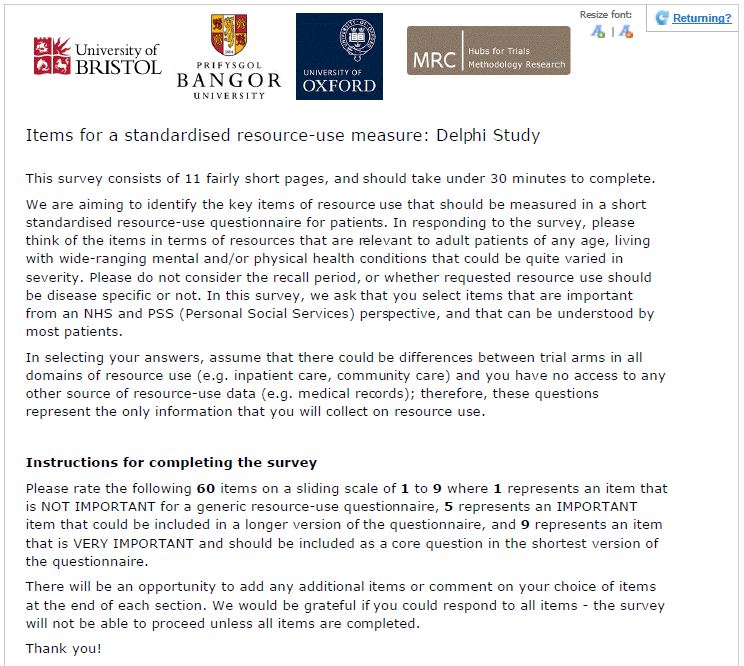


**Figure A2.** Instructions for round 2.


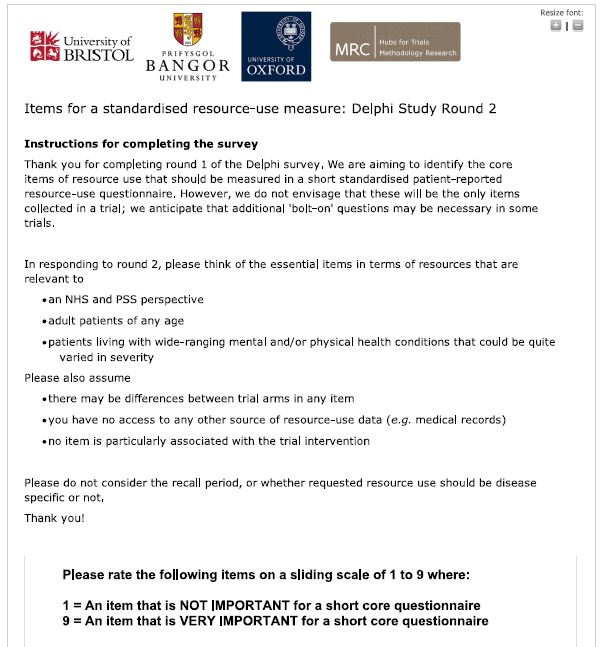

Supplement: Supplementary file 1 — Supplementary material [file mmc1.docx]
